# En Bloc Pancreaticoduodenectomy for Locally Advanced Right Colon Cancers

**DOI:** 10.1155/2017/5179686

**Published:** 2017-07-02

**Authors:** Cihan Ağalar, Aras Emre Canda, Tarkan Unek, Selman Sokmen

**Affiliations:** Department of General Surgery, Dokuz Eylül University School of Medicine, İzmir, Turkey

## Abstract

Locally advanced right colon cancer may invade adjacent tissue and organs. Direct invasion of the duodenum and pancreas necessitates an en bloc resection. Previously, this challenging procedure was associated with high morbidity and mortality; however, today, this procedure can be done more safely in experienced centers. The aim of this study is to report our experience on en bloc right colectomy with pancreaticoduodenectomy for locally advanced right colon cancers. Between 2000 and 2012, 5 patients underwent en bloc multivisceral resection. No major morbidities or perioperative mortalities were observed. Median disease-free survival time was 24.5 months and median overall survival time was 42.1 (range: 4.5–70.4) months in our series. One patient lived 70 months after multivisceral resection and underwent cytoreductive surgery and total pelvic exenteration during the follow-up period. In locally advanced right colon tumors, all adhesions should be considered as malign invasion and separation should not be done. The reasonable option for this patient is to perform en bloc pancreaticoduodenectomy and right colectomy. This procedure may result in long-term survival with acceptable morbidity and mortality rates. Multidisciplinary teamwork and multimodality treatment alternatives may improve the results.

## 1. Introduction

The reported rate of adjacent organ invasion in colorectal cancer is 5.5–16.7% [1, 2]. Compared to other locations, left colonic and rectosigmoid tumors have a higher locally invasive potential [3, 4]. Although tumors located in the right colon have a less invasive potential, surrounding tissue or organ invasion such as duodenum, pancreas, liver, gall bladder, right kidney, and adrenal gland was reported. In locally advanced tumors, it is hard to differentiate neoplastic invasion from inflammatory adhesions intraoperatively. Direct invasion of the duodenum and pancreas may cause surgical difficulties which necessitate an en bloc resection and may discourage the surgeon. Only few series were published on en bloc multivisceral resection with pancreaticoduodenectomy (PD) for right colon cancers. Previously, this challenging procedure was associated with high morbidity and mortality; however, today, this procedure can be done more safely in experienced centers [5, 6]. The aim of this study is to report our experience on en bloc right colectomy (RC) with PD for locally advanced right colon cancers (LARCC).

## 2. Materials and Methods

Between 2000 and 2012, patients who underwent en bloc multivisceral resection (RC and PD) for primary LARCC and had a complete follow-up were included. Patients with primary or recurrent pancreas and duodenum tumors and recurrent colon cancers and those who had peritoneal carcinomatosis or distant metastasis were excluded. When the primary LARCC was adherent to the duodenum and pancreas, we performed en bloc multivisceral resection including RC and PD to achieve clear margins. Patient demographics, presenting symptoms, operation data, histopathological results (tumor diameter, histopathological type and differentiation, depth of invasion, lymph node status, and surgical margins), perioperative morbidity and mortality, and oncological follow-up data were analyzed retrospectively from a prospectively collected database.

In our institution, we discuss and plan treatment of all patients having colorectal malignancy both pre- and postoperatively in multidisciplinary team meetings. We follow up our patients by using the American Society of Colon and Rectal Surgeons (ASCRS) practice parameters for the surveillance and follow-up of patients with colon cancer [7]. We classify complications according to Clavien-Dindo classification [8]. Grade 3 and 4 complications were defined as major morbidity [8]. Mortality occurring during the postoperative hospital stay or within 30 days of operation was recorded as perioperative mortality. Local recurrence was defined as recurrence in the tumor bed, regional lymph nodes, and/or adjacent structures. Distant recurrence was defined as radiologic and/or histologic evidence of a tumor in the parenchyma of any organ, soft tissue, and/or peritoneum (outside of the tumor bed).

## 3. Results

Five patients who had LARCC with invasion to the duodenum and pancreas were included in the study. Patients' demographical characteristics are summarized in Table 1. Three patients presented with anemia and weakness; one patient was diagnosed during routine postoperative follow-up of a rectal adenocarcinoma. Another patient underwent an emergency surgery for acute gastrointestinal bleeding. Locally advanced disease was diagnosed during preoperative staging in four patients who underwent elective surgery and during operation in one patient who underwent emergency surgery. None of the patients had distant metastasis and received neoadjuvant treatment.

All patients had an en bloc RC and PD (one patient underwent total PD). Median operation time was 420 (range: 380–490) minutes. Median intensive care unit (ICU) and hospital stay was 1 and 14 days, respectively. We did not observe major complications including hemorrhage, intra-abdominal abscess, anastomotic leakage, reoperation, or perioperative death. One patient had deep surgical site infection treated by intravenous wide spectrum antibiotics and negative pressure wound management (Case #5). Perioperative data are summarized in Table 1.

All patients had histopathologically proven pT4a tumors invading the duodenum and/or pancreas. Median tumor diameter was 8.5 (range: 6–9) cm. LN metastasis was detected in all patients. Median metastatic LN count was 2 (range: 1–4) and median harvested LN count was 24 (range: 19–38) (Table 1). The surgical margins were negative in all patients except for one who had microscopically positive circumferential margin (Patient #1).

All patients received adjuvant systemic chemotherapy. We observed disease recurrence in four patients. Median disease-free survival time was 24.5 (range: 4.5–38.9) months and median overall survival time was 42.1 (range: 4.5–70.4) months. Patient #1 had retroperitoneal recurrence and underwent surgical exploration unfortunately considered as unresectable because of major vascular invasion. Patient #2 died after a car-pedestrian accident. Patient #3 underwent cytoreductive surgery (CRS) and hyperthermic intraperitoneal chemotherapy (HIPEC) for peritoneal recurrence 25 months after RC and PD. After this procedure, she received systemic chemotherapy. Unfortunately, she developed a pelvic recurrence 35.5 months after CRS and HIPEC procedure and underwent total pelvic exenteration with ileal conduit urinary diversion. She was unable to recover and died 7.5 months after this operation. Patient #4 developed locoregional recurrence invading an ileal segment and underwent en bloc resection 38 months after RC and PD. He died 3 months after this operation after developing a pulmonary thromboembolism. Patient #5 developed liver metastasis 4 months after RC and PD and died during receiving systemic chemotherapy. Oncological follow-up data are summarized in Table 1.

## 4. Discussion

Adjacent organ and/or tissue invasion in locally advanced colon cancer necessitates an en bloc multivisceral resection. The peritoneum (35%), abdominal wall (25%), jejunum and ileum (16%), and omentum (16%) are the most commonly invaded organs or tissues [9]. Right colonic tumors are among the less locally invasive colon cancers. The reported rate of adjacent organ or tissue invasion in right colon cancers is between 11 and 28% [4, 10–13].

Computerized tomography (CT) is a standard tool for preoperatively staging colon cancers. It may give information about T stage, lymph node status, and presence of distant metastasis and has a sensitivity of 86% for detecting tumor invasion [14]. Upper gastrointestinal endoscopy can be performed in suspicious cases, but it should not be forgotten that it could be normal if invasion of the duodenum did not reach the mucosal layer [12].

In 1929, Turner was the first surgeon who described the partial duodenum resection for locally advanced right colon cancer [15]. A series of eight cases was published in 1947 by Calmenson and Black; in this series, all patients had duodenum invasion by LARCC and were treated with right colectomy and partial resection of the duodenum [5]. In 1953, Van Prohaska et al. [16] reported that a patient underwent PD and RC for a locally advanced right colon cancer.

Invasion to adjacent tissues and organs is a sign of aggressiveness in tumor biology but this condition is not always related to distant metastasis [17]. In our series, distant metastasis was not observed in any patient; besides, extended resection is not advised in metastatic patient groups [18, 19]. When the surgeon confronts a LARCC during the operation, all adhesions to the tumor should be considered as malign invasion, since adhesions were reported as 33–84% malignant on pathologic examination [6, 12]. Separation should not be done, because separation of adhesions is associated with high (70–100%) local recurrence rates [6, 17, 20–22]. The nature of the adherence between the primary and adjacent viscera should be determined only upon histologic assessment of the extended resection specimen. If the surgeon does not have enough clinic experience, the suggested modality is to close the abdomen and refer the patient to an experienced center [23]. In our series, we performed en bloc resection in all patients and adhesions were confirmed as malignant in all patients after histopathologic examination.

The risk of morbidity and mortality is another discouraging factor for the surgeon. In standard RC + PPD, the surgeon should do five anastomoses: Wirsungojejunostomy (WJ) or Wirsungogastrostomy (WG), choledochojejunostomy (CJ), gastrojejunostomy, ileotransversostomy, and jejunojejunostomy. Reported anastomotic leakage ratio is 0–33% for any anastomosis in this procedure [22]. In our series, anastomotic leakage did not occur in any patient. All WJ or WG and CJ procedures were performed by hepatopancreaticobiliary surgeons working in our department. This collaboration may explain the zero anastomotic leakage ratio in our series. The reported mortality rate between 0 and 30 days after en bloc pancreas and colon resection is 6.3% [22]. There was no statistically significant difference in perioperative death between only PPD, only RC, and RC + PPD [22]. No perioperative death was seen in our series. LARCC, through* en bloc* PD, has been performed with acceptable mortality and morbidity rates in high volume centers [6, 24]; on the other hand, some authors pointed out the fact that only favorable results in this major surgical technique are published [12]. General suggestion for the patients with comorbidities is to choose partial duodenectomy option if the papilla of Vater is free from invasive disease instead of major surgery [17, 25]. However, this procedure may result in a higher local recurrence rate [22]. In our series, Case #3 deserves to get more attention since it has interesting features such as long-term survival after en bloc RC + PPD despite the high risk of the operation (ASA score was 3). We agree with Patel and Zenilman's opinion which is not to accept the comorbidities as contraindication criteria, if the center has enough clinic experience in extended surgical procedure [26].

Although we did not try to separate the adhesions in any of the patients, one patient (Case #5) had local recurrence in the fourth month. Either neoadjuvant chemoradiotherapy or intraoperative radiotherapy (IORT) can be reasonable options in a patient with locally advanced colon cancer invading into adjacent organs, but there are a limited number of publications in the literature about this issue [27–29]. By using new radiotherapy methods and devices, radiotherapy can be used with lower morbidity by preserving adjacent organs and tissues. A recent study indicates that neoadjuvant chemoradiotherapy combined with en bloc resection may enhance the R0 resection rate and reduce the recurrence rate [27]. Neoadjuvant chemotherapy may also result in downstaging of primary tumor [30]. Another study shows that a patient group that received IORT for locally advanced colon cancer has significantly lower recurrence on tumor bed [29].

Some authors accentuated that lymph node metastasis is one of the most important factors for survival [31–33], besides the other important factor en bloc resection with negative surgical margins. In the literature, it was published that 8 patients underwent R0 en bloc colectomy and PD for locally advanced colon carcinoma and survived nearly or more than ten years (Saiura et al., five patients [6]; Praderi et al., two patients [34]; and Berrospi et al., one patient [23]). The reported 5-year survival after R0, en bloc RC + PD, is 52% [35]; on the other hand, 5-year survival is 0−23% [3, 20, 22, 36] in patients who underwent surgery by separation of adhesions to adjacent organs. Median survival was 42 months in our series; in one extreme case that had two recurrences after RC and PD, she underwent major surgeries for local recurrences (pelvic exenteration and CRS and HIPEC). She was able to survive for about 6 years after RC and PD.

## 5. Conclusion

In conclusion, en bloc RC with PD may result in long-term survival in patients having LARCC with acceptable morbidity and mortality rates. Multidisciplinary teamwork and multimodality treatment alternatives may improve the results.

## Figures and Tables

**Table 1:** Patient demographics, perioperative data, histopathological features and oncological follow up data.

	Patient 1	Patient 2^*∗*^	Patient 3	Patient 4	Patient 5
Age/Gender	55/Male	74/Female	67/Female	58/Male	56/Male
ASA score	2	2	3	2	1
Surgical procedure	En bloc RC and PPD	En bloc RC and PPD	En bloc RC and PPD	En bloc RC and TPD	En bloc RC and PPD
Operation time (min.)	390	380	420	440	490
ICU stay (days)	1	1	1	2	2
Hospital Stay (days)	15	8	14	11	31
Histopathological type	Adenocarcinoma	Adenocarcinoma	Adenocarcinoma	Adenocarcinoma	Adenocarcinoma
Tumor differentiation	High	Low	High	High	High
Max. tumor diameter (cm)	7.5	9	9	8.5	6
Metastatic/total lymph node count	3/21	2/19	4/32	2/38	1/24
Disease free survival (months)	37.9	4.5	24.5	38.9	4.1
Overall survival (months)	60.9	4.5	70.4	42.1	9.1

^*∗*^Underwent emergency surgery; ASA: American Society of Anesthesiologists; RC: Right hemicolectomy; PPD: Proximal pancreaticoduodenectomy; TPD: Total pancreaticoduodenectomy; ICU: Intensive Care Unit.
